# The Three Terms Task - an open benchmark to compare human and artificial semantic representations

**DOI:** 10.1038/s41597-023-02015-3

**Published:** 2023-03-02

**Authors:** V. Borghesani, J. Armoza, M. N. Hebart, P. Bellec, S. M. Brambati

**Affiliations:** 1 Centre de recherche de l’Institut universitaire de gériatrie de Montréal, Montréal, QC H3W 1W6 Canada; 2 Department of Psychology, Université de Montréal, Montréal, QC H3C 3J7 Canada; 3 Department of English, New York University, New York, NY 10003 USA; 4 Vision and Computational Cognition Group, Max Planck Institute for Human Cognitive and Brain Sciences, 04103 Leipzig, Germany; 5 Department of Medicine, Justus Liebig University, 35390 Giessen, Germany

**Keywords:** Human behaviour, Language

## Abstract

Word processing entails retrieval of a unitary yet multidimensional semantic representation (e.g., *a lemon’s colour, flavour, possible use*) and has been investigated in both cognitive neuroscience and artificial intelligence. To enable the direct comparison of human and artificial semantic representations, and to support the use of natural language processing (NLP) for computational modelling of human understanding, a critical challenge is the development of benchmarks of appropriate size and complexity. Here we present a dataset probing semantic knowledge with a three-terms semantic associative task: which of two target words is more closely associated with a given anchor (e.g., *is lemon closer to squeezer or sour?*). The dataset includes both abstract and concrete nouns for a total of 10,107 triplets. For the 2,255 triplets with varying levels of agreement among NLP word embeddings, we additionally collected behavioural similarity judgments from 1,322 human raters. We hope that this openly available, large-scale dataset will be a useful benchmark for both computational and neuroscientific investigations of semantic knowledge.

## Background & Summary

A key aspect of human intelligence is the ability to store and retrieve knowledge on objects, facts, and people, via symbols: reading the word *lemon* activates a multidimensional yet unitary concept which includes its physical attributes (e.g., *a lemon is yellow and roundish*) but also its relations to other concepts (e.g., *you can use a squeezer to get juice out of a lemon*)^1^. Cognitive neuroscience investigations of the behavioural correlates and neural substrates of semantic representations have focused on probing biological agents with carefully designed semantic paradigms and thoroughly selected stimuli, often inferring representational content and structure from semantic judgments on pairs of words^2,3^. Similarly, in natural language processing (NLP), models are often compared against curated benchmarks using behavioural data as ground truth^4^.

However, while NLP models progressively approximate human-like language performance, it is increasingly challenging to evaluate the nature of their internal representations and how closely they align with those supporting human understanding. Virtually all currently used benchmarks, i.e., a task and its related dataset of stimuli and responses, suffer from one or more of the following limitations (Table 1). First, they are rather limited in size, typically offering not more than a thousand stimuli. For instance, WordSim-353, a dataset including pairs of words linked by either semantic similarity (*cup-mug*) or semantic relatedness (*cup-coffee*)^5^, contains only 353 word pairs^6^. SimLex-999, a dataset specifically targeting semantic similarity, includes a total of 999 pairs^7^. The size of the stimuli dataset is critical to enable future applications in settings with data-hungry models^8^.

**Table 1:** Commonly used similarity-based benchmarks.

#	Task Name	# of Words Pairs	Kind of Words Pairs	Measure Provided	# of Raters per pair	Ratings	Reference	
1	8-8-8	8**	nouns	aggregate	8	detect the outlier	Camacho-Collados and Navigli, 2016	^27^
2	MC	30	nouns (subset of RG)	aggregate	38	5-point scale from 0 to 4	Miller and Charles, 1991	^55^
3	RG	65	nouns	aggregate	51	5-point scale from 0 to 4	Rubenstein and Goodenough, 1965	^56^
4	YP-130	130	verbs	aggregate	6	5-point scale from 0 to 4	Yang and Powers, 2006	^9^
5	Verbs-143	143	verbs	aggregate	10	10-point scale from 1 to 10	Baker *et al*., 2014	^57^
6	WS-353-REL	252	nouns + verbs	aggregate and individual score	13 or 16*****	11-point scale from 0 to 10	Agirre *et. al*., 2009	^5^
7	WS-353-SIM	203						
8	MTurk-287	287	nouns, verbs, adjectives	aggregate	10	5-point scale from 1 to 5	Radinsky *et al*., 2011	^58^
9	WS-353	353	nouns + verbs	aggregate and individual score	13 or 16	11-point scale from 0 to 10	Finkelstein *et al*., 2001	^6^
10	SemEval-2017	500*	nouns	aggregate	3	5-point scale from 0 to 4	Camacho-Collados *et al*., 2017	^59^
11	WikiSem500	500***	nouns	aggregate (and only of a subset)	6	detect the outlier	Blair *et al*., 2016	^26^
12	MTurk-771	771	nouns + verbs	aggregate and individual score	(at least) 20	5-point scale from 1 to 5	Halawi *et al*., 2012	^60^
13	SimLex-999	999	nouns, verbs, adjectives	aggregate	36	11-point scale from 0 to 10	Hill *et al*., 2014	^7^
14	SCWS	2003****	nouns, verbs, adjectives	aggregate and individual score	10	11-point scale from 0 to 10	Huang *et al*., 2014	^61^
15	Rare-Word	2034	nouns, verbs, adjectives	aggregate and individual score	10	11-point scale from 0 to 10	Luong *et al*., 2013	^12^
16	MEN	3000	nouns + verbs	aggregate	50	relative comparison to other pairs	Bruni *et al*., 2014	^62^
17	SimVerb-3500	3500	verbs	aggregate and individual score	10	7-point scale from 0 to 6	Gerz *et al*., 2016	^63^

Fifteen benchmarks available in the field of Natural Language Processing to investigate semantic representations with similarity-based tasks. Benchmarks are sorted by size (i.e., number of word pairs available). Notes: (*) each monolingual dataset (English, German, Spanish, Italian, Farsi) has 500 pairs, the size of the multilingual varies from 912 (Italian-German) to 978 (English-Spanish); (**) 8 clusters of 8 related words and 8 outliers each; (***) at least 400 cluster for each language, each cluster with at least 7 related words and 3 outliers; (****) words are presented in sentences, 241 pairs are same-word pairs (e.g., ready to pack his bags vs. another pack of zombies); (*****) same as in WS-353, manually split by two annotators.

Second, available benchmarks have typically undergone minimal behavioural validation (e.g., surveying about 10 raters), and they often offer only aggregate measures (e.g., average scores over all the raters). For example, ratings of semantic closeness were collected in 6 volunteers for the verbs pairs in YP-130^9^, and in 10 for the nouns and verbs pairs in WS-353^6^. Providing fine-grained information on human performance is critical if the goal is that of approximating (or learning more about) the neuro-cognitive substrate of semantic representations in humans^10,11^.

Third, most benchmarks targeting semantic representations have used similarity or association-based tasks comparing two words at a time, often including rather common and frequently used words. While explicit attempts have been made to cover more complex and less frequent words^12^, the overwhelming majority of these datasets does not cover the breadth and depth of human semantic knowledge. Recently, researchers at Google and OpenAI have launched Beyond the Imitation Game Benchmark (BIG-bench, https://github.com/google/BIG-bench)^13^, a collaborative multi-task benchmark to probe large language model performance. Most of the tasks included cover aspects of language such as syntax and grammar, but some tap into semantic knowledge as they require to determine similarity among words and concepts. While taking a major step forward, most of the tasks included in this collection lack behavioural validation, and the stimulus sets are relatively small (for a recent survey of word embedding evaluations via word semantic similarity task, see^14^).

Overall, thus, the field would benefit from large and appropriately validated benchmark datasets to enable fair comparison of artificial and human semantic representations^15^. Such benchmarks would not only foster the improvement of computational models but also be an instrumental tool in empirical investigation of the neuro-cognitive correlates of semantic representations (e.g.^16^).

Here, we present a large-scale benchmark probing semantic knowledge with an associative task: an anchor term (e.g., *lemon*) is associated with two candidate targets (e.g., *squeezer, sour*). We built a total of 10,107 triplets, for a total of 6,433 unique words, including both abstract and concrete words, and spanning not only various semantic categories but also a broad range of length, frequency of use and familiarity, imaginability, and age-of-acquisition. For 2,555 triplets we provide a human ground-truth: the choices made by at least 17 human raters, randomly pulled from a total of 1,322 evaluators.

## Methods

### Semantic task design

The task is modelled after common neuropsychological tests of associative semantic knowledge, such as the Pyramids and Palm Trees Test (PPTT; Howard & Patterson, 1992) and the Camel and Cactus test^17^. Semantic representations are probed by presenting three English words and asking to determine which of the two target words is closer in semantic space to the anchor, hence the name: Three Term Task or 3TT. Please note that in this setting no explicit distinction is made between relations based on features similarity (e.g., *cup – mug*^18^) vs. associative links (e.g., *cup - coffee)*, often further broken down into, for instance, domain vs. function similarity^19^. Contrary to previous tasks relying on the direct comparison of pairs of words/concepts, we require simultaneous comparison of three elements, which has two benefits. First, this task promotes deep semantic processing: different features and dimensions (e.g., real-world size, prototypical location, associated movement) need to be considered at the same time. Second, triplets allow for minimal context effect: e.g., given the word *coffee, cup* will be considered the right choice if the alternative is *plate*, but the choice would change if the other candidate is *bean*. Finally, the forced-choice procedure provides a method that is free of drifts in response criterion within participants, eliminating differences between participants caused by different use of scales (as would be the case for explicit similarity judgments on a Likert scale).

Alternative tasks that have been used to compare distributional model of semantic knowledge include: (a) synonym detection^20^, i.e., given one word (e.g., *levied*) choose the appropriate synonym among four candidates (e.g., *imposed, believed, requested, correlated*); (b) concept categorization^21,22^, i.e., given one word (e.g., *screwdriver*) choose the appropriate taxonomic category (e.g., *tools*); (c) selection preference^23,24^, i.e., how plausible a given noun (e.g. *bike*) is as subject/object of a verb (e.g. *ride*); (d) analogy^25^, i.e., solve problems of the form *“A is to B as C is to?”*; and (e) outlier detection^26,27^, i.e., given a set of words, identify the one not semantically associated with the group.

It should be noted that a benchmark that is useful for computational linguistics does not necessarily respond to the needs of empirical investigations in cognitive neuroscience of language and semantics. As human representations have been shown to be complex and multidimensional^28^, any task loading disproportionally on one aspect or the other would be limiting. For instance, synonyms detection and concept categorization tasks over-emphasize the above-mentioned taxonomic reactions (e.g., *cup - mug*) which are better captured by feature-based models (i.e., focusing on the number of sensory-motor features shared). On the contrary, selection preference tasks stress thematic reactions (e.g., *drink - cup*) which are better captured by distributional models looking at word co-occurrence and relative frequencies. Overall, similarity-based tasks (including analogies resolutions) are the best proxy for the depth and breadth of human semantic processing: as long as proper behavioural data and an adequate sample size are provided, the aspects of these tasks that have been previously criticised become their strengths^29^.

### Triplets generation

To generate the triplets, we developed in-house code (see Code Availability) requiring, at minimum, three inputs: the list of words to be used as anchor, words concreteness ratings, and a pre-trained word embedding to be used to define word distances. Our anchor words were the 1,854 concepts from the THINGS database^30^, given their comprehensive semantic coverage of nameable concrete objects. Concreteness ratings were taken from the crowdsourcing norming study conducted by Brysbaert and colleagues on 37,058 generally known English lemmas^31^, and the pre-trained word embedding selected was a fasttext model trained on WikiNews (1-million-word vectors trained on Wikipedia 2017, UMBC web-based corpus, and statmt.org news dataset; 16B tokens^32^).

The code can accommodate multiple triplet templates each defining different criteria for each word (anchor, potential target 1, potential target 2). To generate our triplets, we defined two templates: (1) all three words need to be nouns with a concreteness rating higher than 4.5 (n = 10,000), thus selecting only concrete nouns; or (2) all three words need to be nouns with a concreteness rating higher than 1, thus *also* selecting abstract nouns (n = 2,000).

**Fig. 1:**
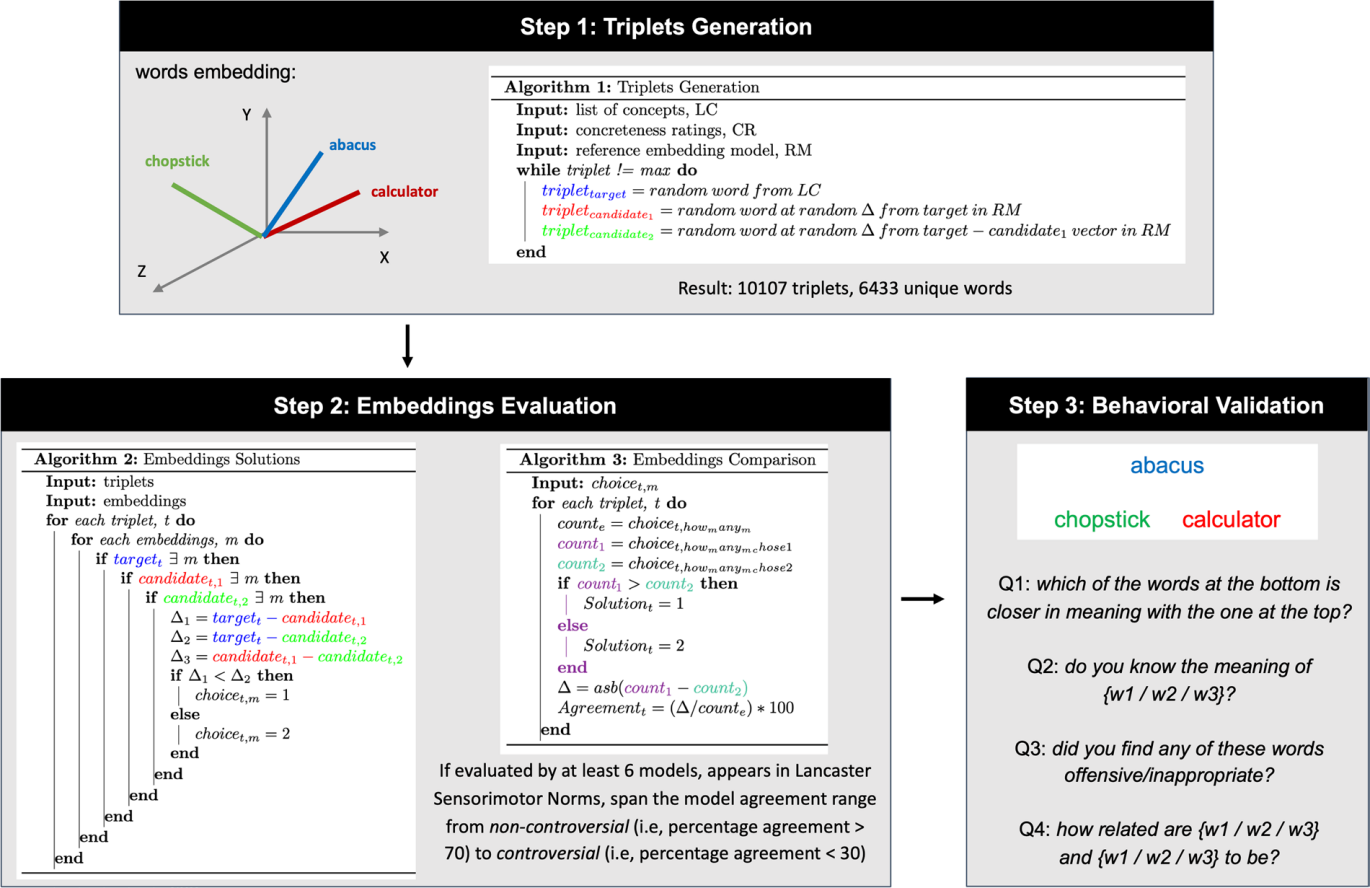
Schematic overview of the step of the benchmark creation. The first step was the generation of 10107 triplets (6,433 unique words). The second step was the collection of the solution chosen by the different NLP embeddings models (n = 14) and the calculation of the NLP embeddings consensus response. The third and final step was the behavioural validation of a subset of triplets (n = 2,555).

Here we briefly describe the sampling strategy for both anchors and candidate targets (Fig. 1 - step 1, algorithm 1). Necessarily arbitrary choices were made with one goal in mind: to sample widely the semantic space, producing a large collection of diverse triplets. Note that unused or less frequently used words are prioritised by tracking the number of times a given word is used, and the method used to define proximity in a given word embedding model is the 3CosMul^33^ method adopted by gensim (https://tedboy.github.io/nlps/generated/generated/gensim.models.Word2Vec.most_similar_cosmul.html). For each template provided, the anchor word is selected from the source list given the template filtering criteria (e.g., all three words need to be nouns with a concreteness rating higher than 1), using the following steps:

1. A first attempt is made, strictly adhering to the criteria provided.
2. If an appropriate word is not found, a second sampling attempt tries to locate the most concrete noun from the anchor source list.
3. Finally, if that fails, an anchor word is randomly chosen among the unused or least used candidate words that have already been sampled for previously generated triplets.

To select the first of two candidate target words, we look at words closest to the anchor word in the embedding model that match that candidate’s filter criteria, specifically:

1. Randomly select a minimum (n_min_) and maximum (n_max_) distanced index from the anchor in that list of words, n_min_ words away or n_max_ words away, respectively.
2. Randomly choose the closer or further list index and choose a random word within a set delta = 5 words around that index.

Then, to select the second of two candidate target words:

1. Calculate the vector formed by the difference between the anchor and first candidate word vectors in the embedding model;
2. Produce a list of words closest to that vector in the model;
3. Constrain the list based on the filter criteria for the second candidate word.
4. Attempt to randomly pick a word;
5. If no words match these criteria, attempt to choose the most concrete noun;
6. If that fails, choose a random word from the list of candidate words already used in previously generated triplets.

Finally, all triplets are randomly shuffled so the different types defined by the filters in the template are intermixed.

**Fig. 2:**
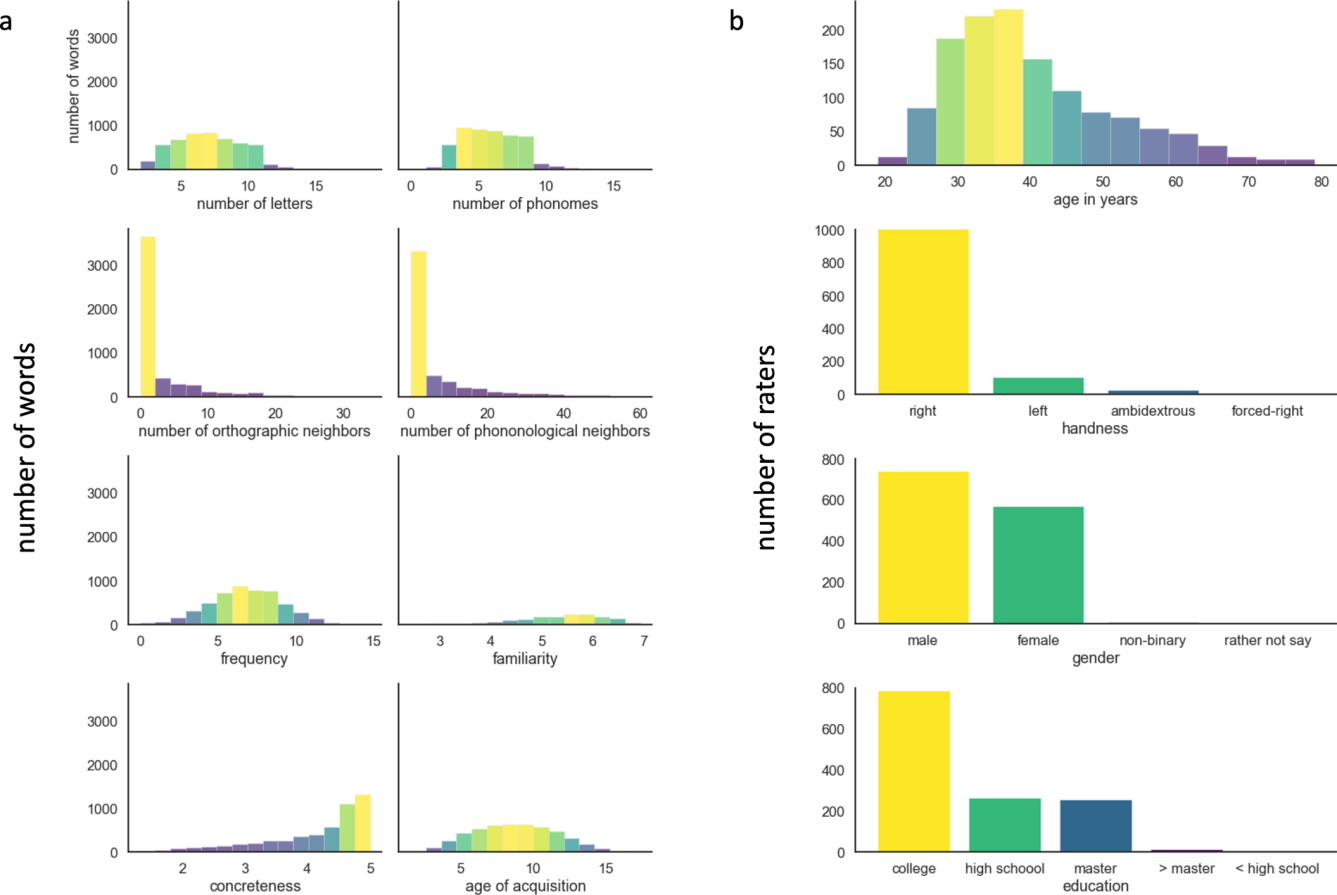
Key stimuli and raters information. Distribution of basic neuropsychological variables across all the unique words included (**a**) and of basic demographic information on the raters (**b**).

The resulting 12,000 triplets were cleaned from duplicates (1,635), and cases where the same word appeared as anchor and target word 1 (n = 175) or anchor and target word 2 (n = 66). Hence, the final dataset included 10,107 triplets, for a total of 6,433 unique words. The full list of triplets can be found in **Triplets_10107.csv**, while the list of unique words, along with their main psycholinguistic variables derive from the South CarOlina Psycholinguistic mEtabase (SCOPE^34^). are in **Triplets_unique_words.csv**. For each word, we also included its synsets in WordNet^35^, and then, for each synset, the synonyms, holonyms, meronyms, hyponyms, hypernyms, and entailments. These psycholinguistic variables, known to affect word form (e.g., number of letters), lexical (e.g., frequency of use), or semantics (e.g., concreteness) aspects of word processing, can be used to sub-select the triplets depending on the use-case (see Usage Note section). Some key variables are reported in Table 2: number of letters (ranging from 2 to 19, mean 7.06, std 2.33), number of phonemes (ranging from 0 to 17, mean 5.81, std 2.06), number of orthographic neighbours (ranging from 0 to 7.5, mean 2.51, std 1.13), number of phonological neighbours (ranging from 0 to 9.5, mean 2.45, std 1.28), frequency of use (ranging from 0 to 14.79, mean 2.30, std 2.30), familiarity (ranging from 2.42 to 6.94, mean 5.33, std 0.89), concreteness (ranging from 1.33 to 5, mean 4.11, std 0.84), age-of-acquisition (ranging from 1,58 to 17.4, mean 8.78, std 2.78). Please note that not all measures are available for all words (Fig. 2a).

**Table 2:** Psycholinguistic properties of the words included in the benchmark.

	Number of Letters	Number of Phonemes	Orthographic Neighbors	Phonological Neighbors	Frequency	Familiarity	Concreteness	Age of Acquisition
#	5,156	5,156	5,156	5,156	5,156	1,604	5,144	4,878
mean	7.06	5.82	2.51	2.46	6.76	5.33	4.12	8.78
std	2.34	2.07	1.13	1.28	2.3	0.9	0.84	2.78
max	19	17	7.5	9.5	14.79	6.94	5	17.4
min	2	0	0	0	0	2.42	1.33	1.58
25%	5	4	1.75	1.55	5.25	4.8	3.67	6.67
50%	7	6	2.45	2.25	6.79	5.5	4.46	8.75
75%	9	7	3.3	3.35	8.35	6	4.76	10.84

We report mean, standard deviation (std), higher and lower values (max, min), as well as 25, 50, and 75 percentiles of eight key variables known to affect word form (i.e., number of letters and of phonemes), lexical (i.e, orthographic neighbours, phonological neighbours, frequency of use), or semantics (i.e., concreteness, familiarity, age of acquisition) aspects of word processing.

### NLP Embeddings selection

NLP word embeddings have become a central tool for understanding the semantic relationship between different words and how this relates to behaviour and the brain (e.g.^36^). To evaluate how these models would fare in the 3TT dataset, we chose embeddings derived from different NLP models and trained on different corpora (Table 3). It should be noted that, as a result, the embeddings do not share the same reference semantic space, i.e., a given word might be present in one but missing in another.

**Table 3:** Key characteristics of the NLP word embeddings included in the benchmark.

#	Name	Model	Trained on	Dimensions	Tokens	Vocabulary size	Reference	
0*	WikiNews	fasttext	Wikipedia 2017, UMBC corpus and statmt.org news	300d	16B tokens	1 M	Mikolov *et al*., 2017	^32^
1	WiGi50d	GloVe	Wikipedia 2014 and English Gigaword Fifth Edition	50d	6B tokens	400k	Pennington *et al*., 2014	^37^
2	WiGi100d	GloVe	Wikipedia 2014 and English Gigaword Fifth Edition	100d	6B tokens	400k	Pennington *et al*., 2014	^37^
3	WiGi200d	GloVe	Wikipedia 2014 and English Gigaword Fifth Edition	200d	6B tokens	400k	Pennington *et al*., 2014	^37^
4	WiG300d	GloVe	Wikipedia 2014 and English Gigaword Fifth Edition	300d	6B tokens	400k	Pennington *et al*., 2014	^37^
5	WiG300d42B	GloVe	Common Crawl	300d	42B tokens	1.9 M	Pennington *et al*., 2014	^37^
6	Twitter25d	GloVe	Twitter (2B tweets)	25d	27B tokens	1.2 M	Pennington *et al*., 2014	^37^
7	Twitter50d	GloVe	Twitter (2B tweets)	50d	27B tokens	1.2 M	Pennington *et al*., 2014	^37^
8	Twitter100d	GloVe	Twitter (2B tweets)	100d	27B tokens	1.2 M	Pennington *et al*., 2014	^37^
9	Twitter200d	GloVe	Twitter (2B tweets)	200d	27B tokens	1.2 M	Pennington *et al*., 2014	^37^
10	Reddit	sense2vec	Reddit comments 2019			3.3 M	Trask *et al*., 2015	^38^
11	CCsub	fasttext	Common Crawl	300d	600B tokens	2 M	Mikolov *et al*., 2017	^32^
12	Amazon	fasttext	Amazon reviews 2016			1.4 M	Joulin *et al*., 2016a; Joulin *et al*., 2016b	^39,40^
13	Yahoo	fasttext	Yahoo answers 2016			1.9 M	Joulin *et al*., 2016a; Joulin *et al*., 2016b	^39,40^
14	Yelp	fasttext	Yelp reviews 2016			500k	Joulin *et al*., 2016a; Joulin *et al*., 2016b	^39,40^

For each embedding used in the current study, we include the underlying model (i.e. fasttext, glove, sense2vec), the corpora it was trained on, and its dimensions. The index 0* denotes the embedding model used to generate the triplets, not included in the following analyses.

In total, fourteen different embeddings were selected. Five GloVe^37^ models trained on Wikipedia 2014 and English Gigaword Fifth Edition, four with 6B tokens yet different dimensions - 50,100,200, and 300d respectively, and one with 42B tokens and 300d. Four additional GloVe models trained on Twitter with different dimensions - 25,50,100,200d respectively. A sense2vec^38^ model trained on Reddit comments. A fasttext^32^ model trained on Common Crawl with subword information. Finally, three additional fasttext^32,39,40^ models trained on Amazon reviews, Yahoo answers, and Yelp reviews respectively.

### NLP Embeddings comparison

Would NLP word embeddings agree on the solution to a given triplet? To answer this question, we examined each triplet across all 14 embeddings with the following steps. First of all, for each embedding, we checked that all three words were present in the embedding-specific semantic space, otherwise, that embedding was skipped for that particular triplet. Second, we assessed the distance in the embedding-specific semantic space between anchor and target word 1 (*Δ*1), anchor and target word 2 (*Δ*2), and target word 1 and target word 2 (*Δ*3). The solution chosen by each embedding was then determined by the comparison of *Δ*1 and *Δ*2 (Fig. 1 - step 2, algorithm 2). Finally, two measures were considered at the group level: which target words had been selected by most of the embeddings (NLP word embedding consensus choice) and how strong was the agreement across embeddings. The agreement index was calculated by first computing the delta between the number of embeddings choosing target word 1 and those choosing target word 2 and then computing the percentage of this value relative to the total number of embeddings tested for that triplet (Fig. 1 - step 2, algorithm 3). For instance, the triplet [anchor: *gurney* - target word 1: *ambulance* - target word 2: *dishtowel*] could be evaluated only in 6 embeddings, which split equally between the two target words leading to an agreement of 0%. In contrast, the triplet [anchor: *mallet* - target word 1: *chainsaw* - target word 2: *tambourine*] could be evaluated in all 14 embeddings, all of which selected the target word 1 leading to perfect agreement (100%, see Table 4 for more examples).

**Table 4:** Examples of various triplets and their solution according to NLP embedding models and human raters.

Examples of	Anchor	Target 1	Target 2	# models choosing 1	# models choosing 2	% agreement	# raters choosing 1	# raters choosing 2	% agreement
“hard” triplets for NLP embeddings	arrow	pellet	toolbox	7	7	0	1	25	92.3
	chandelier	ballroom	candlestick	7	7	0	13	13	0
	abacus	chopstick	calculator	7	7	0	2	22	83.33
	coffeemaker	kitchenette	thermos	7	7	0	13	12	4
	broom	fern	janitor	7	7	0	6	22	51.14
	sheep	alpaca	people	7	7	0	23	3	76
“easy” triplets for NLP embeddings	mallet	chainsaw	tambourine	14	0	100	14	5	47.37
	candle	lamp	candlelight	0	14	100	10	23	39.39
	cream	ice	lavender	0	14	100	27	1	92.86
	radio	broadcaster	telephonic	0	13	100	20	2	81.81
	ship	deck	courier	0	12	100	19	5	58.33
	fire	flood	charcoal	0	12	100	3	28	80.65
Same anchor, different targets	trolley	carousel	grocery	7	7	0	22	7	51.72
	trolley	monorail	farmhouse	4	10	42	19	3	72
	trolley	railway	lollipop	1	13	85	30	2	87.5
	trolley	sidewalk	ejector	1	12	84	22	5	62.96
	trolley	streetcar	basket	1	13	85	25	7	56.25
	trolley	streetcar	shelf	6	8	14	32	1	93.94

The first 6 rows present triplets that are controversial for NLP models (no consensus decision can be reached), while the following 6 rows list triplets where NLP models perfectly agree. The last 6 rows illustrate cases of triplets with the same anchor but different target words 1 and 2. The human raters’ choices are listed on the right as a comparison.

### Online behavioural testing

Does the NLP word embedding consensus choice match the solution chosen by human raters? To answer this question, a subset of triplets was selected for behavioural validation. We chose those triplets that (1) had been evaluated by at least 6 of the above-mentioned embeddings, to ensure reliable coverage of the NLP models semantic space; (2) appeared in Lancaster Sensorimotor Norms^41^, a database including 39,707 concepts rated along 11 sensory-motor dimensions, to ensure wider future adoption in cognitive neuroscience; (3) spanned the model agreement range to ensure inclusion of both *non-controversial* and highly *controversial triplets*^42^. Non-controversial triplets show high agreement between embeddings, such as for [anchor: *mallet* - target word 1: *chainsaw* - target word 2: *tambourine*] for which all 14 embeddings chose target word 1. While *controversial triplets* are those that are solved differently by NLP embeddings, for instance, given the anchor [*arrow*], seven embeddings chose the target word [*pellet*] while the other seven the target word [*toolbox*]. Of the total of 2,555 triplets submitted for behavioural validation, 17.85% were *non-controversial* (i.e, percentage of agreement >70, n = 456), while 54.64% were *controversial* (i.e, percentage of agreement <30, n = 1396).

Among the selected triplets, there were a total of 3,630 unique words (1,041 unique anchor, 1,908 unique target word 1, 2,011 target word 2). Of the unique words used as anchors, 211 appear as such in at least 4 triplets, allowing the study of minimal context effect. Table 4 illustrates examples of triplets with high and low NLP embedding agreement, as well as examples of triplets sharing the same anchor. The full list of triplets that underwent behavioural validation can be found in **Triplets_behavioral_2555.csv**.

We generated one png image per selected triplet, with the anchor always being displayed on the top while randomising whether the target word would appear on the left or right side of the image (Fig. 1 - step 3). A Qualtrics (Qualtrics 2020; https://www.qualtrics.com) survey was built and distributed via the crowdsourcing platform Amazon Mechanical Turk (Mturk^43^, https://www.mturk.com) between May and November 2021. All experimental procedures complied with the *Centre de Recherche Institut Universitaire de Gériatrie de Montréal (CRIUGM) Ethics Committee* and the *Centre intégré universitaire de santé et de services sociaux du Centre-Sud-de-l’Île-de-Montréal* requirements (*CÉR-VN: Comité d’Éthique de la Recherche- Vieillissement et Neuroimagerie*), in line with the principles expressed in the Declaration of Helsinki. Ethical approval was obtained before the start of the study (CER VN 20-21-29), and all participants read and agreed to the corresponding informed consent. Participants received monetary compensation for their participation. Raters were required to be physically located in the US or Canada, have a MTurk approval rate greater than 96% and have a number of MTurk tasks approved higher than 50. Moreover, before beginning the experiment, raters were asked to provide basic demographic details: gender, age, educational level, hand preference, country of origin, country of residence, native language, and any other language spoken. The total number of raters involved in the study was 1,322. Each triplet was evaluated by a variable number of raters ranging from a minimum of 17, to a maximum of 49, with a mean of 25.63 and standard deviation 4.39 (the full information is stored in Response_Summary.csv, column number_of_resp).

Raters were then presented with one triplet at a time and asked to determine which of the words at the bottom was closer in meaning to the word at the top (Fig. 1 - step 3). Immediately afterwards, they were asked to declare whether they knew the meaning of each of the three words or not. They were also offered the chance of flagging a given word as offensive or inappropriate. Finally, they were asked to indicate how close in meaning they perceived each pairwise combination of the three words on a continuous scale (with no labels attached, but implicitly converted to a 0-to-9 scale). Raters were instructed to provide the first answer that came to their mind, attempting a guess whenever needed. Raters were asked to evaluate 45 triplets, but they were free to interrupt testing at any given time.

As for the word embeddings (Fig. 1 - step 2, algorithm 3), two measures were considered at the group level: which target words had been selected by most of the raters (human raters consensus choice) and how strong was the agreement across raters. The agreement index was calculated by computing the delta between the number of raters choosing target word 1 and those choosing target word 2, and then computing the percentage of this value relative to the total number of raters that evaluated that triplet. For instance, the triplet [anchor: *abacus* - target word 1: *chopstick* - target word 2: *calculator*] was rated by 24 humans, 22 of which selected target word 2, thus leading to 83.3% agreement index [calculate via: (22-2)/24*100]. We also quantified how easy each triplet was as the delta between the average similarity between anchor and target word 1 vs. anchor and target word 2 as rated on the continuous scale: the closer the two similarities are, the harder to adjudicate between the two targets. Following the previous example, the average similarity between abacus and chopstick was 2.25, between abacus and calculator 7.08, thus making this an easy triplet to solve. This situation can be compared with the triplet [anchor: *jean* - target word 1: *pant* - target word 2: *denim*], rated by 36 humans who chose target 1 15 times, and target 2 21 times (agreement index = 12.67%): in this case the average similarity between *jean* and *pant* was 7.41, between *jean* and *denim* 7.66.

## Data Records

All data are available at https://osf.io/at8cs/^44^. The data repository contains the full dataset (all the triplets generated and basic descriptors of the unique words included) as well as the behavioural dataset (the subset of triplets for which human behavioural ratings were collected). The same repository also includes the supporting documentation (e.g., the ethics committee of CRIUGM and CÉR-VN approval letter), the analysis code prepared for this study, and all related metadata (e.g., subjects’ instructions). The file **variables_descriptions.md** helps navigating the different columns of each datasets described below.

### Full dataset

The full 3TT dataset includes the set of 10,107 triplets generated (**Triplets_10107.csv**) along with detailed information on each of the 6,433 unique words they contain (**Triplets_unique_words.csv**). We provide several neuropsychological and linguistic variables of interest, such as number of letters, frequency of use, familiarity, concreteness, imaginability, age-of-acquisition, as available from South CarOlina Psycholinguistic mEtabase (SCOPE^34^). Moreover, for each word, we also included its synsets and then, for each synset, the synonyms, holonyms, meronyms, hyponyms, hypernyms, and entailments as per WordNet^35^.

### Behavioural dataset

The 3TT behavioural dataset includes four csv files. First, the 2,555 triplets (for a total of 3,630 unique words) that have undergone behavioural validation (**Triplets_behavioral_2555.csv**).

Second, basic demographics and performance information on the raters (**Results_Demographics_1322.csv**). It should be noted that the two tables, containing raw anonymized individual subject data (i.e., participant demographics, Results_Demographics_1322.csv, and individual’s ratings for each stimuli, Results_Responses_1322.csv) will be accessible only after registration with the CNeuromod databank (https://docs.cneuromod.ca/en/2020-alpha2/ACCESS.html) due to ethical considerations. These data can be used to decide to exclude given raters based on their performance and/or characteristics: **RandID** ensure raters identification while preserving their anonymity. For each rater we report: gender, age, education, handedness, native language, country of origin, current country, whether they speak languages other than English. We also include information on mean (and standard deviation) of the reaction times (in second), total test time (in minutes), number of “*not sure I know the word*” and *“the word is possibly offensive*”.

Third, the raw yet anonymized responses for each of the 2,555 triplets and 1,322 raters (**Results_Responses_1322.csv**), and the summary of the results (**Results_Summary.csv**), where for each triplet we report a number of statistics regarding the identity of anchor and target words, the number of responses collected for each, the choice, percentage of agreement, response times (mean and standard deviation), as well as the mean and standard deviation of all the pairwise distances between three items as rated on the continuous scale.

## Technical Validation

### NLP Embeddings comparison

On average, for a given triplet 12.48 (std 2.77) out of the 14 NLP embeddings could be evaluated, since not all words were present in all NLP embeddings. While 2 triplets could not be covered by any NLP embeddings, these triplets are still included as other or possibly future embeddings might include all relevant words. Overall, across embeddings, the agreement was 49.38% (std: 23.75%), ranging from perfect (100%) to null (0%). In 6,808 triplets, the two target words were more similar to each other than to the target in at least half of the embeddings they were compared on. For instance, given [anchor: *bag* - target word 1: *pharmacy* - target word 2: *lump*], the two target words are judged closer to each other than either of them to the anchor by 13 (out of 14) NLP word embedding models.

### Online behavioural testing

We collected responses from 1,322 MTurk workers (Fig. 2b) Of these, 742 identified as male, 570 as female, 6 as non-binary, and 3 preferred not to say. There were 1,189 right-handers, 103 left-handers, 26 ambidextrous, and 3 forced right-handers. Mean age was 39.62 years, std 11.29, ranging from 19 to 79 years old. Mean education was 15.62 years (conversion adopted: high school/GED = 12, college’s degree = 16, master’s degree = 18, Ph.D. = 20) with std = 1.99. Given our criteria, raters were located either in the USA (n = 1,306), or in Canada (n = 15). The countries of origin were the USA in the overwhelming majority of the cases (n = 1,248), with additional 16 raters from Canada and the remaining 57 raters coming from a range of 36 different countries. While all were fluent in English, 334 raters spoke at least one other language, with 32 cases in which the native language was other than English: Arabic (n = 4), Armenian, Finnish, French, German, Hindi (n = 3), Korean, Mandarin (n = 3), Nepali, Persian, Romanian, Russian, Spanish (n = 10), Swahili, Tamil. All triplets were judged by at least 15 raters, but users of the benchmark might decide to screen responses based on specific criteria (see section Usage Note).

**Fig. 3:**
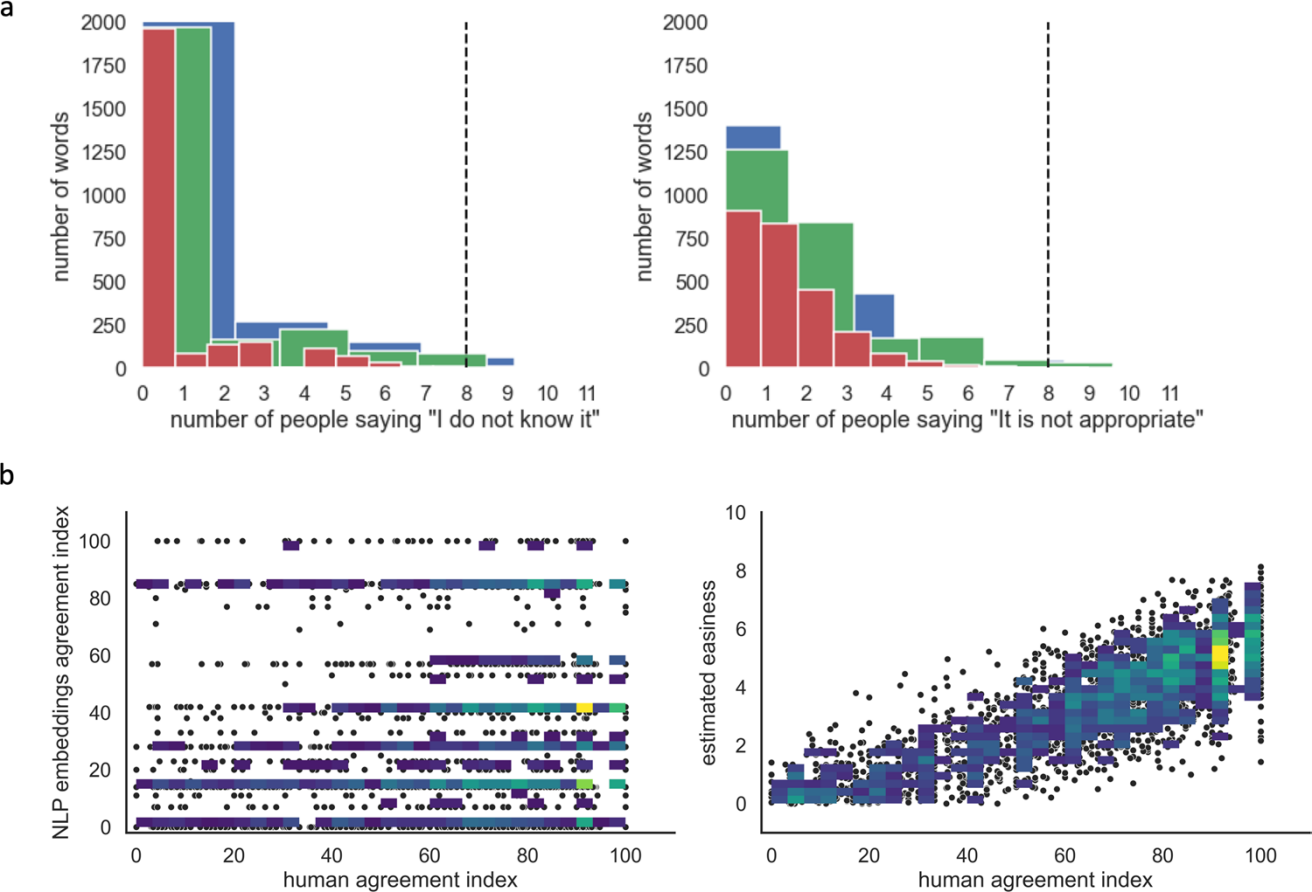
Behavioural validation of the dataset. (**a**) Histogram of the words declared as unknown (left) or judged as offensive (right), the dotted line at the number 8 indicates the 98th percentile of the distribution. (**b**) Scatterplot of the human agreement index vs. the NLP embedding agreement index (left) and of the human agreement index vs. the estimated easiness in solving the triplet, defined as the absolute distance between the two candidate targets (right).

Here, we report basic analyses supporting the technical quality of the dataset. (All steps can be found in the supporting notebook ColabNotebook_3TT.) First, we excluded triplets that contained words judged as offensive (n = 30) or declared as unknown (n = 68) by more than 8 raters. This arbitrary threshold was chosen after inspecting the histogram of all responses and observing that it fell on the 98th percentile of the distribution (Fig. 3a). Thus, a total of 2,457 triplets entered further analyses.

The selected triplets were rated by an average of 25.46 raters (std 4.25, min = 17, max = 42), and the overall agreement index was 64.28% (std: 26.47), ranging from perfect (100%) to null (0%). As it can be appreciated in Fig. 3b on the left, both human and NLP embedding agreement spanned the whole range from very high to very low. Unsurprisingly, human agreement correlated with the estimated easiness in solving the triplet, defined as the absolute distance between the two candidate targets (r = 0.8, Fig. 3b, on the right). It should be noted that the wide range of human agreements allows for a double dissociation between triples hard to solve by human consensus vs. by NLP embedding models’ consensus. For instance, [anchor: *coffeemaker* - target word 1: *creamer* - target word 2: *hairdryer*] appears to be a controversial triplet for NLP embeddings (8 vs 6 split), but not for humans (90% agreement on target word 1). Similarly, triplets might be controversial for humans but not NLP embeddings: for example, given [anchor: *candle* - target word 1: *lamp* - target word 2: *candlelight*] human raters are split (10 chose target word 1, 23 target word 2), while the choice of embeddings is unanimous (14 for target word 2).

Considering only the subset for which the models reach a consensus (n = 2137), the solution chosen by the majority of human raters overlapped with the NLP embeddings’ choices only for 21.15% of the triplets. Conversely, human raters and the neuro-cognitive inspired model Lancaster Sensorimotor Norms agree 74.54% of times. It should be noted that this does not mean that any single NLP embedding is anti-correlated with the human raters. Rather, trying to reach a consensus decision based on the embeddings here included leads to a very poor performance.

**Fig. 4:**
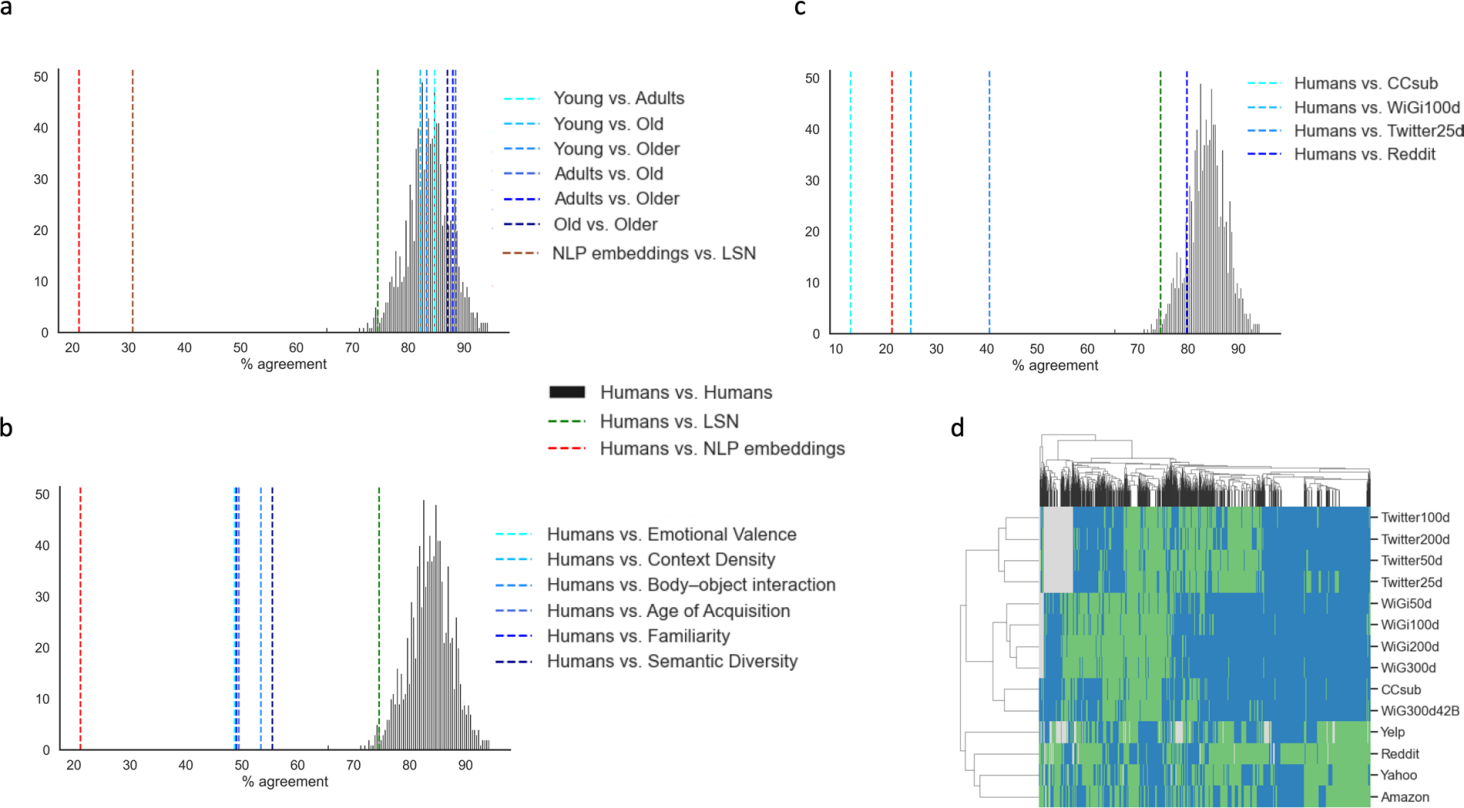
Assessment of the benchmark. (**a**) Percentage of agreement of solutions based on the consensus among NLP embeddings (red), the Lancaster Sensorimotor Norms (green), as well as the pairwise comparison of different demographic groups (blue scale). The four demographic groups are defined as 4 demographic groups: young (<30 years, n = 286), adults (30–40 years, n = 501), old adults (40–50 years, n = 279), older adults (>50 years, n = 251). The black histogram indicates agreement levels between randomly assigned groups of human raters (200 raters per group, procedure repeated 1,000 times). (**b**) As in (a) but showing the percentage of agreement of solutions based on single measures capturing one lexico-semantic aspect at a time. (**c**) As in (a) but showing the percentage of agreement of 4 representative NLP embeddings (the best, the worst, and two intermediate ones). (**d**) Hierarchically clustered heatmap of the NLP embeddings (rows) based on their responses to each triplet (columns).

We investigated potential age effects by splitting the human raters in 4 demographic groups: young (<30 years, n = 286), adults (30–40 years, n = 501), old adults (40–50 years, n = 279), older adults (>50 years, n = 251). To create a null distribution of the agreement level between human raters, we randomly assigned participants to two groups of 200 raters and computed their agreement, repeating the procedure 1,000 times. The results indicate an average agreement of 83.63% (std = 3.88), ranging from 65.28% to 94.31%. In Fig. 4a, we illustrate how all age groups fall well between the distribution of the random splits of raters, while the sensorimotor norms results fall below the 2 percentiles, and the NLP embeddings are outside of the distribution support.

As a final sanity check, we evaluated how other measures capturing only one lexico-semantic aspect at a time would fare: emotional valence (i.e., the pleasantness of a stimulus^45^), contextual diversity (i.e, how many different passages a word is found in^46^), body-object interaction (i.e., the ease with which the human body can interact with a words’ referent^47^), age of acquisition^48^, familiarity^49^, and semantic diversity (i.e., the variance in context associated with a given word^50^). All these variables fell short of sensorimotor norms, with performances around chance level: emotional valence (48.67%, n = 900), context density (48.95%, n = 1,667), body-object interaction (53.41%, n = 1,084), age of acquisition (49.49% n = 1,580), familiarity (49.03%, n = 1,703), and semantic diversity (55.47%, n = 759) (Fig. 4b).

To further illustrate the potential of this dataset as a benchmark for NLP models, we present the results for each of the 14 NLP embeddings here considered. The overall ranking indicates that the sense2vec model trained on Reddit is the best at approximating the human ratings (Table 5), the only one outperforming the sensorimotor norms results (Fig. 4c). It should be noted that the Reddit model is not only the most recent and with the largest vocabulary, but it is also the only sense2vec model included. Compared to glove and word2vec models, it allows senses disambiguation (e.g., bank as a financial establishment vs. piece of land alongside water^38^). Overall, it appears that a combination of training dataset, vocabulary size, and model kind determines the ability to match human choices. First, fasttext models outperform glove ones, except for the fasttext model trained on Common Crawl. As a matter of fact, the two models trained on the web archive achieve the lowest performance (WiG300d42B and CCsub). Second, glove models trained on Twitter achieve better results than those trained on Wikipedia. Third, given the same model, training set, and vocabulary size, fewer dimensions seem to lead to better results. Figure 4d further illustrates the clustering of NLP embeddings according to their responses to each triplet.

**Table 5:** Ranking of the NLP word embeddings.

Name	Agreement with Humans	
	# of triplets	percentage
Reddit	1960	79.77
Yahoo	1424	57.96
Amazon	1301	52.95
Yelp	1148	46.72
Twitter25d	996	40.54
Twitter50d	880	35.82
Twitter100d	786	31.99
Twitter200d	729	29.67
WiGi50d	652	26.54
WiGi100d	612	24.91
WiGi200d	560	22.79
WiG300d	546	22.22
WiG300d42B	434	17.66
CCsub	317	12.90

For each embedding used in the current study, we computed the number of triplets for which there is agreement with the human ratings and the corresponding percentage.

Overall, we believe these analyses demonstrate that our task and dataset are a good benchmark as they are (1) accurately and unambiguously annotated reflecting human semantic representations, (2) of sufficiently large size as to allow deployment in several machine learning settings, (3) controversial enough to make it difficult (or impossible) for NLP embedding to converge on one solution. We show that NLP embeddings are still far from reaching human-like semantic representations and thus saturating this benchmark, while neuro-cognitive oriented models taking into account sensory-motor information can successfully guide such endeavours^51^. It should be noted that sensory-motor information alone is not sufficient to reach inter-human level of agreement, suggesting a margin for improvement of neuro-cognitive models as well, perhaps complementing the experiential information they cover with distributional information^28^.

Finally, it should be noted that our unique datasets included both abstract and concrete terms, which have been shown to be cognitively and neurally dissociable^52^, thus opening the way to studies addressing, for instance, the role of experiential and distributional information in warping the semantic distance between these two different words classes.

## Usage Note

The full dataset can be freely downloaded from https://osf.io/at8cs/. We also provide a notebook to perform basic exploration of the dataset and the analysis here reported: ColabNotebook_3TT. It should be noted that the two tables containing raw anonymized individual subject data (i.e., participant demographics, Results_Demographics_1322.csv, and individual’s ratings for each stimuli, Results_Responses_1322.csv) will be accessible only after proper registration with the CNeuromod databank (https://docs.cneuromod.ca/en/2020-alpha2/ACCESS.html) due to ethical considerations.

Data provided in **Results_Demographics_1322.csv** can be used to remove raters based on, for instance, their native language, country of origin, or performance. We would recommend against using reaction times as the collection of such a measure through MTurk is inherently noisy. Instead, we would suggest removing triplets in which at least one word has been judged offensive or was not known by 8 or more raters.

We believe that this dataset will be useful, in years to come, to compare NLP models of word meaning with human semantic representations. As noted by Schnabel and colleagues^53^, NLP embedding models rankings might vary depending on the benchmark chosen. In particular, item-based evaluations (e.g., pairwise similarities) might diverge from set-based evaluations (e.g., intrusion task), suggesting that (1) global measures are necessary to shed light into the differences between embeddings, and (2) the more benchmarks the better.

Here we focused on word vector models, which have been shown to learn relationships between words as they are deployed in the corpora they are trained on. One might wish to extend the study to large language models, for instance quantifying semantic similarity between human raters and different layers of GPT3. While the rise of large language models holds promise for models reaching human-like language performance, it remains difficult to evaluate whether what is learned by these models aligns with human understanding. Are they memorising shallow linguistic information or accessing human-like representational knowledge? Currently, contextual, experiential information captured by norms such as LSN is missing from virtually all NLP models: our observations might guide the enhancement of these models and our dataset will be the perfect benchmark for such efforts.

## Data Availability

The code used to generate the triplets and compare the embeddings is made available at https://osf.io/at8cs/. The code to generate triplets [code/compare_triplets] requires, at a minimum, three inputs: the list words to be used as anchor, words concreteness ratings, and the pre-trained embedding to be used to define word distances. This code can be used to generate novel triplets fitting other experimental goals, for instance triplets at fixed distances between target words or triplets with only abstract (or concrete) terms. The code to compare embeddings [code/generate_triplets] requires the generated triplets and the embeddings one wishes to use to solve the triplet task. It can easily be adapted to test novel embeddings (e.g., with different training samples or vocabulary sizes). We also provide a notebook to perform basic exploration of the dataset including all the analysis here reported: ColabNotebook_3TT (available on the OSF repository as well). All analysis can be reproduced with the data directly available on the OSF repository safe for the two requiring individual subject data: Results_Demographics_1322.csv and Results_Responses_1322.csv will be accessible only after proper registration with the CNeuromod databank (https://docs.cneuromod.ca/en/2020-alpha2/ACCESS.html) due to ethical considerations. The OSF repository includes also the datasheet for the dataset^54^: https://osf.io/echny. Data and code are released under Creative Commons Attribution 4.0 International Public Licence (CC-BY 4.0^44^).
